# Germination and Growth Characteristics of *nud* Knockout and *win1* Knockout Barley Lines under Salt Stress

**DOI:** 10.3390/plants13091169

**Published:** 2024-04-23

**Authors:** Elena V. Antonova, Nadezhda S. Shimalina, Anna M. Korotkova, Ekaterina V. Kolosovskaya, Sophia V. Gerasimova, Elena K. Khlestkina

**Affiliations:** 1 Institute of Plant and Animal Ecology (IPAE), Ural Branch of Russian Academy of Sciences, 8 Marta 202, Ekaterinburg 620144, Russia; nadia_malina@mail.ru; 2 N.I. Vavilov All-Russian Institute of Plant Genetic Resources (VIR), Bolshaya Morskaya 42–44, Saint Petersburg 190000, Russia; korotkova@bionet.nsc.ru (A.M.K.); kolosovskaya@bionet.nsc.ru (E.V.K.); gerson@bionet.nsc.ru (S.V.G.); director@vir.nw.ru (E.K.K.); 3 Institute of Cytology and Genetics (ICG), Siberian Branch of Russian Academy of Sciences, Prospekt Akad. Lavrentjeva 10, Novosibirsk 630090, Russia

**Keywords:** *Hordeum vulgare*, CRISPR/Cas, *NUD*, *WIN1*, knockout line, salinity stress

## Abstract

*Hordeum vulgare* genes *NUD* (*HvNUD*) and *WIN1* (*HvWIN1*) play a regulatory role in cuticle organization. Because the cuticle is a key evolutionary acquisition of plants for protection against environmental factors, a knockout (KO) of each gene may alter their ability to adapt to unfavorable conditions. A potential pleiotropic effect of *HvNUD* or *HvWIN1* gene mutations can be assessed under salt stress. Initial developmental stages are the most sensitive in living organisms; therefore, we evaluated salt tolerance of *nud* KO and *win1* KO barley lines at the seedling stage. Air-dried barley grains of the KO lines and of a wild-type (WT) line were germinated in NaCl solutions (50, 100, or 150 mM). Over 30 physiological and morphological parameters of seedlings were assessed. Potential pleiotropic effects of the *HvNUD* gene KO under salt stress included the stimulation of root growth (which was lower under control conditions) and root necrosis. The pleiotropic effects of the *HvWIN1* gene KO under the stressful conditions manifested themselves as maintenance of longer root length as compared to the other lines; stable variation of most of morphological parameters; lack of correlation between root lengths before and after exposure to NaCl solutions, as well as between shoot lengths; and the appearance of twins. Salt tolerance of the analyzed barley lines could be ranked as follows: *nud* KO > *win1* KO ≈ WT, where *nud* KO lines were the most salt-tolerant. A comparison of effects of salinity and ionizing radiation on *nud* KO and *win1* KO barley lines indicated differences in tolerance of the lines to these stressors.

## 1. Introduction

In the context of global climate change, one of the important issues in modern agricultural ecology is soil salinization [1]. This process can have natural causes or occur secondarily in irrigated agriculture; soil salinization is one of the factors exacerbating desertification [2]. Anthropogenic activities have accelerated the processes and timeframes (and increased the scale) of salt flows and altered their direction, thereby creating an anthropogenic salt cycle [3]. According to the Global Soil Salinity Map (GSASmap), currently more than 424 million hectares (over 4.4%) of upper (0–30 cm) and 833 million hectares (over 8.7%) of lower (30–100 cm) soil horizons are affected by salinization. More than two-thirds of such soils are located in arid deserts (37%) and steppes (27%) [4]. Therefore, under salinity conditions, plants can also be affected by a water deficit. Osmotic and ionic stress inhibits the growth and development of plants, thus leading to significant crop losses [5].

At present, there are almost no economically viable methods for combating soil salinization; hence, the best practice is to cultivate crops having high salt tolerance [6]. Barley, the fourth most important cereal crop in the world [7], has high salinity tolerance [8,9] as compared to other dominant cereals: wheat (moderately tolerant), rice (sensitive), and maize (moderately sensitive) [10]. One possible way to increase crop yield, plant material quality, and agricultural plant resistance to biotic and abiotic factors is gene editing using the CRISPR/Cas system [11]. Currently, however, rice is the most frequently used model for understanding the mechanisms of cereals’ resistance to biotic and abiotic stressors via genome-editing-based approaches [12].

Genes *NUD* (nudum) and *WIN1* (wax inducer 1) of barley *Hordeum vulgare* (hereafter: *HvNUD* and *HvWIN1*, respectively) are close homologs and encode AP2/ERF-type transcription factors of the *WIN1*/*SHN1* subfamily, functionally associated with the regulation of cuticle organization. Despite their close relatedness, these genes are responsible for different functions. A knockout (KO) of the *HvNUD* gene leads to the transformation of hulled to naked barley [13]. Plant mutants in the *HvWIN1* gene are characterized by a deficiency of epicuticular wax load on the surface of leaves, stems, and spikes in the generative phase of development [14]. The cuticle is considered a key evolutionary acquisition in terrestrial plants and protects them from various abiotic and biotic factors such as high and low temperatures, drought, UV radiation, salinity, and the impact of bacteria, fungi, and viruses [15]. 

Numerous studies have been published showing a pleiotropic effect of *WIN1*/*SHN1* subfamily genes on various properties of plant cuticles [16,17]. Described functions of genes *NUD* and *WIN1* are related to grain formation processes because these functions determine the assembly of the cementing layer between the lemma and pericarp or the production of the wax coating on the outer surface of the lemma, respectively. In this context, the question of the pleiotropic effect of these genes and the tolerance of *nud* KO and *win1* KO lines to abiotic and biotic stressors, including salinity, at the seedling stage, remains relevant.

The laboratory seed germination method is widely used to assess the impact of individual environmental factors (e.g., low or high temperatures, salinity, moisture deficiency, ionizing radiation, heavy metals, nanoparticles, or magnetic fields) or their combined effects by means of biometric parameters (e.g., seed germination energy, seed vigor, seedling survival, and root and shoot lengths) at early stages of plant development [18,19,20,21,22,23,24,25,26]. In several studies, the roll culture method has been applied [19,20,25,27] or Petri dishes have been used [18,21,22,23,28]. In these cases, experiments are conducted in distilled water, aqueous solutions, soil extracts, and suspensions with the adverse factors. The advantage of the roll culture method—involving filter paper and a dense frame made of vellum (tracing paper)—lies in the use of up to 50–100 seeds per replicate, a uniform distribution of roots inside the roll, and the natural vertical position of seedlings [29]. Petri dishes create stable microclimatic conditions for germination, and evaporation of the working solution is minimized due to the cover. Other researchers employ vessels with soil mixtures [21,30]. An advantage of this seed germination method has been established in a study on root growth and development during evaluation of the quality of soil contaminated with heavy metals [31] as compared to soil extracts. The choice of a seed germination method for wild and cultivated plants depends on research objectives, characteristics of seeds of a particular plant species (e.g., hardseededness, the germination rate, and analyzed parameters), and the duration of the experiments.

During the first 1–3 weeks of germination (the timing depends on the plant species)—before the formation of first leaves and the onset of photosynthesis—seedlings utilize reserve substances of the seed and do not require additional nutrition; therefore, experiments are often conducted in distilled water [22,32]. In general, early stages of plant ontogenesis are considered the most vulnerable to various environmental stressors because plants’ subsequent survival directly depends on their response [33]. The nutrient reserves stored in the seed (nonstructural carbon, nitrogen, and phosphorus) are readily available to the developing seedling, in contrast to unpredictable acquisition of nutrients from soil and availability of light [34].

Currently, there are almost no data on the impact of genes *NUD* and *WIN1* on the early stages of plant development. Therefore, the aim of this study was to investigate potential effects of *HvNUD* and *HvWIN1* KOs in barley; these modifications theoretically can influence a complex trait such as salt tolerance. KO lines serve as ideal models for researching effects of gene mutations, including pleiotropic effects [35,36]. Suitable previously generated KO lines called *nud* [13] and *win1* [14] were chosen here as models for assessing salt tolerance. We hypothesized that the KO lines would differ in salt tolerance judging by growth characteristics as compared to a wild-type (WT) barley line (control). Additionally, we were interested in whether the variance in morphological traits increases under the stress in both WT and KO barley lines, and whether the observed responses to salt stress are consistent with those documented in these lines after acute γ-irradiation [28]. In this article, we adhere to the classic interpretation of the terms “stressor” (as a factor of influence) and “stress” (as the organism’s responsive reaction to the impact of a stressor).

## 2. Results

### 2.1. Seed Germination, the Survival Rate, and the Number of Seedlings with Leaves in nud KO and win1 KO Lines under Salinity Stress 

**Figure 1:**
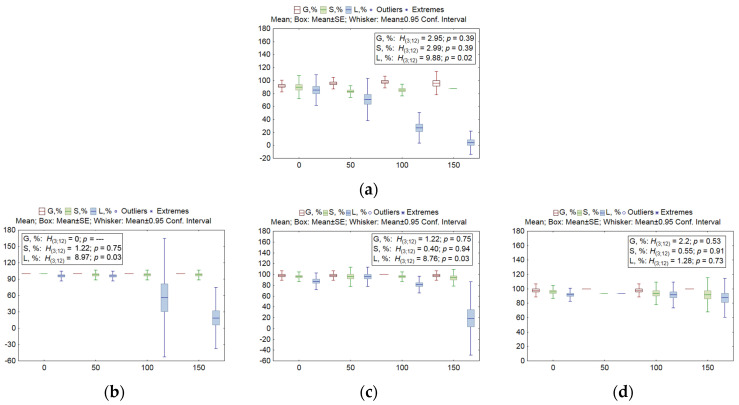

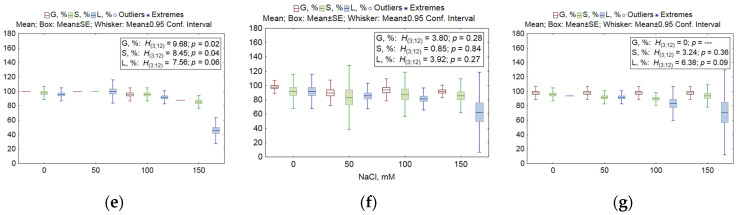
Variation in seed germination (G, %), in the survival rate (S, %), and in the number of seedlings with leaves (L, %) in KO barley lines called *nud* and *win1* after salt stress. (**a**) The WT, (**b**) *nud 07-1*, (**c**) *nud 05-4*, (**d**) *nud 01-4*, (**e**) *win1 25-2-18*, (**f**) *win1 25-2-2*, and (**g**) *win1 17-4-14*. The number of samples per line was 3. Mean values, standard error (SE), a 95% confidence interval, outliers, and extremes are indicated in the figure. Because no outliers and extremes were detected for G, S, and L, such data points are not presented in the figure. The nonparametric Kruskal–Wallis (KW) test was used, where *H* is the KW criterion, and *p* is the significance level. Degrees of freedom (*df*) of the numerator and denominator of *H* are given in parentheses.

Seed germination is one of the most crucial processes in the field of plant biology and ecology because it determines the timing and scale of seedling emergence in each growing season, thereby influencing community dynamics [37]. Characteristics initially evaluated here were seed germination, seedling survival, and the number of seedlings with leaves. The exposure to NaCl solutions with concentrations ranging from 50 to 150 mM did not have a significant impact on seed germination and seedling survival (Figure 1) in all lines (*H*_3;12_ = 0.401–3.23; *p* = 0.284–0.94). An exception was a KO line called *win1 25-2-18*, where these metrics decreased by 12.5–12.8% at the highest salt concentration (*H*_3;12_ = 8.45–9.68; *p* = 0.0215–0.0376). 

A more pronounced response to the salt stressor was observed in the number of seedlings with leaves (see Figure 1). Significant changes at 150 mM (compared to 0 mM) were noted in the WT line (a pairwise comparison using Dunn’s test, *z* = 2.89, significance level: *p* = 0.023) and in the *nud 05-4* KO line compared to 50 mM (*z* = 2.83, *p* = 0.029). Thus, the lines most sensitive to salinity were WT, *nud 07-1* KO, and *nud 05-4* KO: the number of seedlings with leaves decreased, and the salt tolerance index (*STI*) was 4.9%, 19.6%, and 21.4%, respectively. Because the *STI* of the *nud 01-4* KO line was 95.5%, it was the most salt-tolerant based on the number of seedlings with leaves.

### 2.2. Seedling Root and Shoot Growth Parameters of nud KO and win1 KO Lines under Salinity Stress

**Figure 2:**
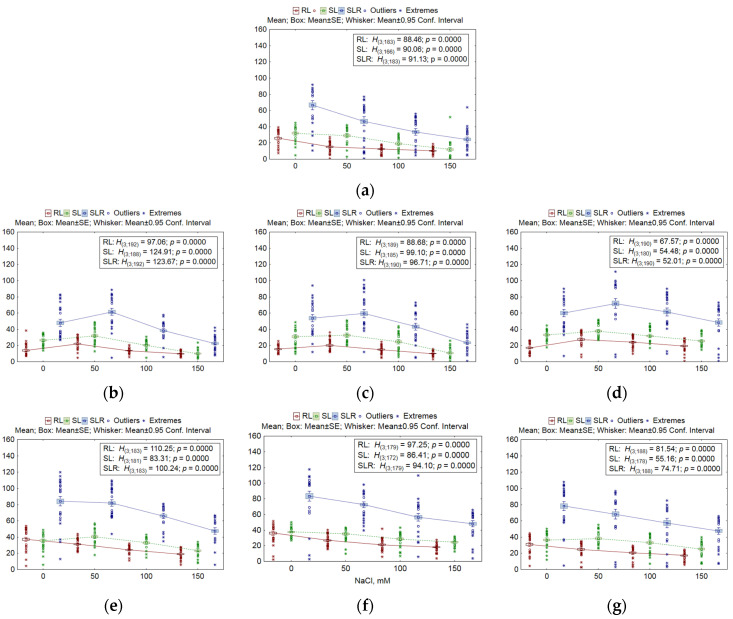
Root length (RL, mm), shoot length (SL, mm), and seedling length (shoot + root; SRL, mm) of *nud* KO and *win1* KO barley lines after salt stress. (**a**) The WT, (**b**) *nud 07-1*, (**c**) *nud 05-4*, (**d**) *nud 01-4*, (**e**) *win1 25-2-18*, (**f**) *win1 25-2-2*, and (**g**) *win1 17-4-14*. The number of samples per concentration or line was 41–48. Mean values, standard error (SE), a 95% confidence interval, outliers, and extremes are indicated in the figure. The nonparametric Kruskal–Wallis (KW) test was used, where *H* is the KW criterion, and *p* is the significance level. Degrees of freedom (*df*) of the numerator and denominator of *H* are given in parentheses.

The parameters studied next were root and shoot length and the total length of seedlings. Unstressed (intact) *nud* KO lines are known to have lower dry weight and shorter root length in comparison with intact *win1* KO lines [28]. Salt solutions significantly affected the growth of shoots and roots of barley seedlings in the seven studied lines (Figure 2). Nonetheless, at the lowest NaCl concentration, shoot length of all lines did not differ from that of intact plants (*z* = 0.29–2.14, *p* = 0.19–1.0). Further increases in NaCl concentration gave differences between the lines. For instance, at the highest salt concentrations, the most sensitive lines were the WT, *nud 07-1* KO, and *nud 05-4* KO, with *STIs* ranging from 25.7% to 33.7% (*z* = 8.34–8.41, *p* << 0.001). In the WT line, a correlation was found between the length of roots before and after exposure to NaCl solutions, and the same was true for shoot length (*R* = 0.33–0.38; *p* = 0.0218–0.0287). Both before and after exposure to salt stress, significant positive associations were observed between root length, shoot length, and total seedling length in all *nud* KO lines (*R* = 0.309–0.499; *p* = 0.0006–0.0364) but only in one *win1* KO line (*win1 25-2-18*; *R* = 0.294–0.375; *p* = 0.0094–0.045). 

Next, the number of roots in seedlings and the total length of the root system were determined. The number of roots almost did not change under the influence of the salt stressor. By contrast, some KO lines (*nud 01-4* and *win1 25-2-18* KO) experienced a stimulatory effect (*STI* = 105.5–111.8%) at a NaCl concentration of 50 mM (*z* = 2.66–3.86, *p* = 0.0007–0.047), whereas at 150 mM (WT and *nud 07-1* KO lines), root formation decreased (*STI* = 83.8–87.9%; *z* = 3.36–4.11, *p* = 0.0002–0.005). 

A stimulatory effect on *nud* KO lines was also noted in terms of the total root length of seedlings and in the analysis of the length of each root individually. In *nud 01-4* KO, this effect (*STI* = 121.5–160.3%) was detectable under the influence of NaCl concentrations of 50–100 mM (*z* = 4.25–9.03, *p* << 0.00013), whereas in *nud 05-4* KO and *nud 07-1* KO (*STI* = 105.4–153.4%), the effect was observed only under the action of the weakest salt stressor (*z* = 2.87–7.46, *p* < 0.024). In the WT line, growth processes were found to be suppressed at all NaCl concentrations (*STI* = 35–56.6%; *z* = 4.15–9.31, *p* << 0.0002), and in *win1* KO lines, either there was no significant response (*p* > 0.049) or the *STI* ranged from 47.7% to 74.8% (*z* = 5.57–7.65, *p* << 0.001). Additionally, there were similar patterns: suppression of growth processes (*STI* = 36.6–70.1%) in the WT line across the entire NaCl concentration range (*z* = 3.7–9.04, *p* << 0.001) as well as stimulation of growth processes (*STI* = 120.3–140.9%) in *nud* KO lines (*z* = 3.43–3.49, *p* = 0.004–0.0019) and no response in *win1* KO lines at 50 mM NaCl in the analysis of the variation in the total seedling length, where shoot and root lengths were summed (see Figure 2).

The ratio of root/shoot length is an important biological parameter that characterizes the competition between upper and lower organs for various nutrients. The shoot provides a plant with carbon, while the root supplies water and nutrients [38]. Because, under normal conditions, the roots of *nud* KO lines were shorter than those of the other lines, and the average shoot length did not differ among lines [28], ratios of average shoot length/total root length and of average shoot length/root length in *nud* KO lines were 1.6–1.8 times higher than those in the WT line and 2 times higher than the ratios in *win1* KO lines. In the latter, these ratios reached 80% of those in the WT line. Under salinity stress, we observed stimulatory effects both in the WT line at the lowest NaCl concentration of 50 mM (*STI* = 124.2–127.5%; *z* = 4.38–4.79, *p* << 0.0001) and in *win1* KO lines throughout the concentration range (*STI* = 103.7–142.9%; *z* = 3.57–7.8, *p* < 0.0021). Such effects were not seen in *nud* KO lines: under salinity stress, the shoot/root length ratio diminished. This means that under salinity stress, all lines experienced desynchronization between above-ground and below-ground organs but in different directions. In *nud* KO lines, the reason was the stimulation of root growth with concurrent slowing of shoot growth, whereas in *win1* KO lines and the WT line, roots slowed their growth more steeply than shoots did. Note that the shoot/root length ratio in seedlings of *nud* KO lines under control conditions was higher than that of *win1* KO lines and of the WT, owing to the short roots of *nud* KO lines [28].

One of the characteristics of a variation range (variance in a trait consisting of individual values of a variable arranged in ascending or descending order of a quantitatively expressed trait) is the coefficient of variance (*CV*, %). Because shoot length and lengths of roots 1–11 (if present) were measured in each seedling, we analyzed the variance of these morphological traits using the *CV*. A fold increase in the *CV* is reported only for 150 mM NaCl because the effect was the strongest at this concentration (Table 1). Under salt stress, 18% of seedling responses were characterized by an increase, and 21% by a decrease in variation. In more than half of the responses, the variance did not change. The greatest increase in variation was registered in the WT line (1.6–3.5-fold as compared to unstressed WT plants). Additionally, 36% of the traits of this line manifested increased variation, and no parameters with a decrease in the *CV* were found. The majority (73–91%) of traits in *win1* KO lines under salinity stress featured stable variation, and an increase in the *CV* occurred in only 9–27% of the responses. Only the *win1 25-2-18* KO line also showed a decrease in trait variance (9%). *Nud* KO lines exhibited diverse responses. In *nud 01-4* KO, a uniform distribution between a decrease in the *CV* and an unchanged *CV* was observed. The other two *nud* KO lines showed three classes of responses, with *nud 07-1* KO mostly exhibiting a decrease in trait variance (64%), and *nud 05-4* KO showing an increased or unchanged *CV*.

### 2.3. Seedling Weights of nud KO and win1 KO Lines under Salinity Stress

**Figure 3:**
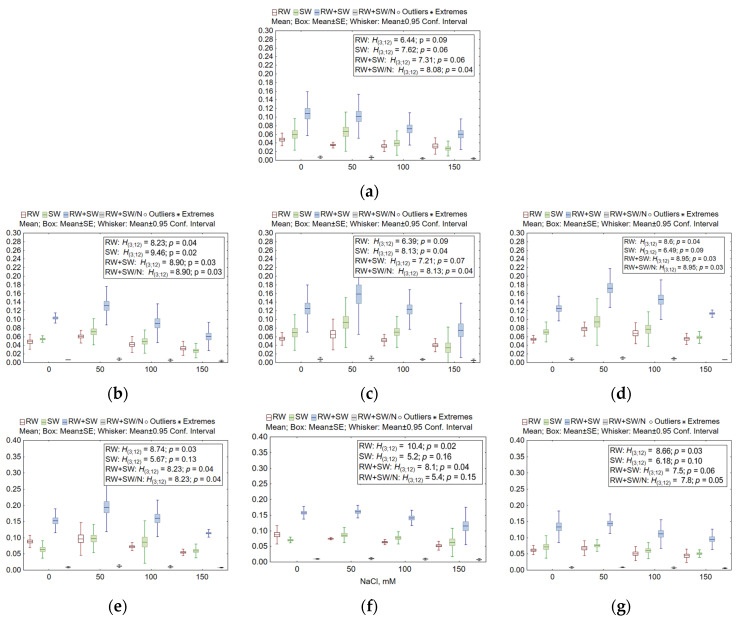
Variation in air-dry weights of seedlings (RW: roots, SW: shoots, RW+SW: seedlings, and RW+SW/N: per seedling) of *nud* KO and *win1* KO barley lines after salt stress. (**a**) The WT, (**b**) *nud 07-1*, (**c**) *nud 05-4*, (**d**) *nud 01-4*, (**e**) *win1 25-2-18*, (**f**) *win1 25-2-2*, and (**g**) *win1 17-4-14*. The number of samples per concentration or line was 3. The mean values, standard error (SE), a 95% confidence interval, outliers, and extremes are indicated in the figure. Given that there were no outliers and extremes detected for RW, SW, RW + SW, and RW + SW/N, such data points are not presented in the figure. The nonparametric Kruskal–Wallis (KW) test was used, where *H* is the KW criterion, and *p* is the significance level. Degrees of freedom (*df*) of the numerator and denominator of *H* are given in parentheses.

Growth characteristics positively correlated with the weight of different organs (*R* = 0.602–0.985; *p* << 0.0001). Nevertheless, the correlation coefficients between the weight of different organs before exposure and after exposure to NaCl solutions were not significant (*R* = −0.99 to −0.0075; *p* = 0.062–0.99). When statistical hypotheses were assessed by the Kruskal–Wallis test (Figure 3), there was no significant effect of salt stress on the weight of roots (WT and *nud 05-4* KO line), shoots (WT and *nud 01-4* KO, and all *win1* KO lines), all seedlings (WT, *nud 05-4* KO, and *win1 17-4-14* KO lines), and per seedling (*win1 25-2-2* KO) of barley. By contrast, pairwise comparisons of root weight at different NaCl concentrations revealed differences between 50 and 150 mM in *nud 07-1* KO (*z* = 2.72, *p* = 0.039) and *win1 25-2-2* KO when groups 0 and 150 mM were compared (*z* = 3.06, *p* = 0.013). Shoot weight differed significantly in two *nud* KO lines (*07-1* and *05-4*) when groups 50 and 150 mM were compared (*z* = 2.83–3.06, *p* = 0.013–0.028). Similar results were obtained for seedling weight in two *nud* KO lines (*07-1* and *01-4*) and in *win1 25-2-18* KO (*z* = 2.77–2.94, *p* = 0.019–0.033). Seedling weight normalized to the number of observations differed between these NaCl concentrations in all *nud* KO lines (*z* = 2.77–2.94, *p* = 0.019–0.033) and in *win1 25-2-18* KO (*z* = 2.83, *p* = 0.028). This finding indicates promotion of the weight of different organs at the weakest salinity stressor: the *STI* in the aforementioned KO lines reached 118.1–137.8%, with suppression of the parameters by 9–50% at the highest NaCl concentration. In total, barley KO lines were more resistant to the salt stressor than the WT line. 

### 2.4. Seedlings’ Abnormalities in nud KO and win1 KO Lines under the Influence of the Salt Stressor

During the study, abnormalities in the development of barley seedlings were discovered, which could be categorized into the following groups (Table 2): (1) present in all lines, (2) absent in all lines, (3) rarely found in the WT, (4) absent in the WT (present in *nud* KO and *win1* KO lines), (5) present only in *nud* KO lines, and (6) present only in *win1* KO lines. Under the action of the salt stressor, an increase in abnormalities of the development of seedlings was noticed, manifesting itself without any obvious patterns. Nonetheless, just as under normal conditions (no salt stress)€ [28], during the NaCl treatment, *nud* KO lines exhibited a change in the shape of roots, reaching 100% prevalence among *nud 07-1* KO seedlings, with root necrosis being seen in 6–20% of seedlings of *nud 07-1* KO and *nud 05-4* KO. For *win1* KO lines, alterations in the shape and color of leaves were observed. An increase in the number of seedlings having root hairiness was observed in all lines under salinity stress.

**Figure 4:**
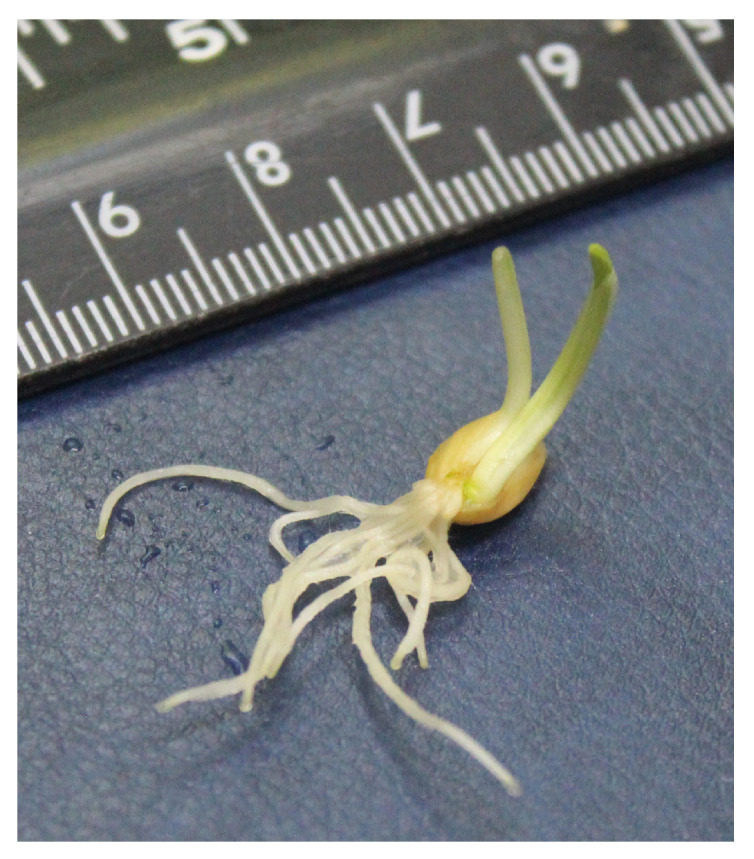
A morphological abnormality found in the *win1 25-2-18* KO line of *H. vulgare*: twins.

It is also worth mentioning that the emergence of twins was noted in *win1 25-2-18* KO line, with a frequency (prevalence) of 6.3% observed under the influence of the weakest salt stressor (Figure 4). Neither in the WT nor in *nud* KO lines was there occurrence of twins. In total, twins had 11 roots, with 6.9 ± 0.61 (mean ± standard deviation) of them in normal seedlings at this salt concentration. All abnormalities of seedling development showed uneven expression along the stressor gradient.

## 3. Discussion

Gross metrics of the KO and control (WT) barley lines, including seed and seedling weight and key morphological parameters at the early [28] and generative [13] stages of plant development, have been described previously. Under normal conditions, the lines did not differ in seed germination, seedling survival, and the number of seedlings with leaves. The *nud* KO lines are known to have lower root weight and shorter length relative to *win1* KO lines under normal conditions [28]. It has been reported that at later stages of development, *nud* KO and *win1* KO lines do not differ from the WT line in key growth characteristics, except for a reduction in 1000-grain weight in hull-less *nud* KO lines, which has been attributed to the absence of lemmas [13,14,28]. 

In the current paper, a short review of salt tolerance data on *H. vulgare* L. cv. Golden Promise (GP) is presented below because the *nud* KO and *win1* KO lines investigated in this work have been derived from this cultivar (Table 3). GP has been obtained through irradiation of cv. Maythorpe and exhibited improved salt tolerance in comparison with parental cv. Maythorpe [39]. At NaCl concentrations (150 mM), GP’s salt tolerance has been reported as moderate [40], whereas at higher concentrations (400 mM), it is deemed low [41]. Another characteristic of cv. GP is the expression of the dwarfing gene, resulting in plants with shorter height as compared to parent cv. Maythorpe [39,42]. Below, we also include a brief overview of naked barley lines and cultivars obtained through natural or targeted mutations. This topic is relevant because naked barley grown in infertile soils often prone to salinity has manifested salt stressor tolerance [43].

Our analysis of published data revealed that the assessment of salt tolerance in naked barley primarily has been focused on molecular, transcriptomic, and biochemical markers [50,51,52,53,54]. There is insufficient information on the weight and length of above- and underground organs in barley seedlings. Experimental studies on the role of transcription factor *HvWIN1* in barley salinity resistance are currently lacking. Using *Brassica napus* as an example, it has been demonstrated that overexpression of the *BnWIN1* gene enhances plant growth and reduces water loss under salt stress conditions [55]. Similar findings are reported for transgenic tobacco [56,57].

Summarizing the data obtained during the present study, we conducted a comparison of 22 responsive reactions to salt stress among KO lines and between the WT line and KO lines (Table 4). Similar tolerance series (highlighted in one color in Table 4) were summed, divided by the total number of responses, and transformed into a percentage. It was demonstrated that the most sensitive responses to salinity (more than 27–31% of reactions) are exhibited by the WT and by *win1* KO lines (Table 5). The most tolerant plants were *nud* KO lines because they exhibited sensitivity in only 9% of reactions and were more resistant than the other lines in >27% of cases. Although nakedness in barley is determined by a mutation in a single gene, this mutation can have widely varied pleiotropic effects due to drastic differences in the physiological state of the embryo. Naked barley has an unprotected embryo; on the one hand, this trait may increase the number of lesions, and, on the other hand, it can increase the likelihood of preadaptation during seed processing and storage. It has been reported that nakedness likely does not exert an epistatic effect on several other loci controlling the expression of traits related to seedling viability [58]. In a study by Hao Chen et al. [50], however, an advantage of naked barley lines over hulled ones was found in terms of seed germination and root and shoot lengths. Stimulatory effects of the weakest salt stressor (in terms of root length and seedling weight; for *win1* KO lines: in the root number) were noted in *nud* KO lines in our work. Similar findings have been obtained by other researchers [59].

According to the literature [60,61,62], a 100 mM NaCl solution does not have a significant impact on barley at early stages of development. At higher concentrations (150–600 mM), weights and lengths of shoots and roots diminish [63,64]. Some barley genotypes at 342 mM did not have germinated seeds [47]. Presumably, concentrations above 150 mM induce plasmolysis in barley, by causing osmotic shock. At low NaCl concentrations (50 mM), osmotic stress occurs, accompanied by moderate changes in cell turgor and osmotic pressure [65]. Differences between the two phenomena also lie in changes of gene expression accompanying osmotic (first) and ionic (second) phases of salt shock or stress. For example, during osmotic shock, high gene expression is detectable within a few minutes after exposure (osmotic phase), whereas during osmotic stress, the overexpression occurs after several days: in the ionic phase [65]. Using sugar beet (*Beta vulgaris* L.) as an example, it has been demonstrated that by leading to the overexpression of a greater number of genes, salt shock induces more significant transcriptomic changes than stress does [66]. The specific reactions of plants to salt shock or stress are also illustrated in a paper on antioxidant defense enzymes [67]. In our study, a biphasic response to the salinity stressor in *nud* KO lines was noted, too, where low concentrations in some cases had a stimulatory effect, whereas high concentrations had a toxic effect. This phenomenon is known as hormesis, which is observed at low doses of radiation or low concentrations of toxicants [68,69,70,71].

In a study by Long’s group [63], a significant correlation between root and shoot weights before and after stress exposure was not observed. Our work yielded similar conclusions regarding these parameters. Other studies also have not detected an association between seed germination and shoot length [47], consistent with our data (*R* = 0.062; *p* = 0.7897). On the other hand, in our experiments assessing the impact of the salt stressor, a correlation between growth characteristics before and after stress exposure was identified in all examined lines, except for *win1 25-2-2* KO and *win1 17-4-14* KO. A significant positive association between initial seedling survival and resistance to additional irradiation has also been found in white campion (*Silene latifolia* Mill.) and smooth brome (*Bromus inermis* Leyss.) under chronic exposure to low doses of ionizing radiation [72,73]. The presence of a significant correlation allows us to predict a plant’s tolerance to any stressors on the basis of viability indicators.

The root/shoot ratio is one of the key ecological parameters for assessing a plant’s ability to compensate for limited resources in the environment and contributes to the organism’s survival [34,74]. Resource allocation between shoots and roots is typically analyzed as a balance between their activities because shoots provide the plant with carbon while roots supply water and nutrients [38]. The root/shoot ratio is also widely used to estimate underground biomass and to calculate carbon reserves in agrological ecosystems [75]. An increase in the root/shoot length ratio, i.e., a decrease in the shoot/root length ratio, is a common response to the salt stressor, and this response is connected to a water deficit and the formation of an osmotic effect rather than a direct action of NaCl [76,77,78]. 

Accelerated root growth under salt stress may contribute to retention of K^+^, Na^+^, and Cl^−^ ions in the roots by reducing their influx into shoots [79]. Nonetheless, some studies have shown that ion concentrations in roots and shoots do not correlate with the rate of root elongation, suggesting that Na^+^ and K^+^ concentrations do not directly influence root growth [46]. Our results indicate that different reactions are possible in barley lines under salt stress, as evidenced by the ratio of above-/underground organs. For instance, *nud* KO lines exhibited stimulated root growth with a slowdown of shoot growth, whereas in *win1* KO and WT lines, roots slowed their growth more steeply than shoots did. Because some studies link the increase in the root/shoot ratio to enhanced salt tolerance [64], *nud* KO lines can be considered the most resistant to salinity judging by this parameter. Additionally, consistently low root/shoot ratios have been registered in seedlings obtained from seeds with greater mass [34].

One of tasks in our study was to investigate the variation of morphological traits under the influence of the salt stressor. The hypothesis we formulated at the beginning of the study—an increase in the variation of traits under stressful conditions—was proven only partially. We observed diverse effects of salt stress on barley lines. Hulled *win1* KO lines featured the stablest variation or even manifested a reduction in this parameter. The decrease in variation and in mean trait values indicated the sensitivity of seedlings to salinity. The variety of observed reactions in the naked lines carrying the *HvNUD* KO points to a possible connection between nakedness and embryonic stress because the embryos are not protected by lemmas. During germination under normal and stressful conditions, this situation causes an overall increase in trait variation. The WT line under salt stress exhibited the greatest increase in variation as compared to intact WT plants. The increase in variation may also be related to different abilities of plants to respond to the stressor at early stages of development. Similar data on standard deviations have been presented by other researchers [22,80]. Additionally, an increase in the variation of root and shoot length has been revealed at the end of experiments in comparison with their beginning [18]. 

The formation of abnormalities in cellular structures and organelles in plants under the influence of different NaCl concentrations is regarded as a relatively common phenomenon [80,81,82], which is associated with biochemical, anatomical, physiological, and other changes. We observed under the action of the salt stressor an increase in abnormalities in the development of seedlings, which manifested itself without any clear-cut patterns. Nevertheless, just as under normal conditions [28], the impact of NaCl on *nud* KO lines was characterized by changes in root shape (reaching 100% prevalence among plants of the *nud 07-1* KO line) and by the emergence of root necrosis in lines *nud 07-1* KO and *nud 05-4* KO. This is likely because in naked lines, the embryo radicle is not covered by lemmas, and, therefore, it is more sensitive to the salt stressor. For *win1* KO lines under salt stress, alterations in leaf shape and color as well as the appearance of twins were observed. The occurrence of twins in the *win1 25-2-18* KO line under stress has been previously documented after γ-irradiation at a dose of 50 Gy [28]. These data imply a role of the *HvWIN1* gene in the formation of embryonic meristems. Morphological abnormalities in the form of organ fusion have been identified in *Arabidopsis* underexpressing genes of the *SHN1*/*WIN1* subfamily [83,84]. It is possible that the KO of the *HvWIN1* gene also affects the embryo cuticle, thereby leading to excessive formation of organs. An increase in the number of seedlings having root hairs was observed in all lines here under the stressful conditions.

Earlier, a comparison of responses of these KO lines to different stressors revealed that fewer developmental abnormalities arise in barley seedlings under salt stress as compared to γ-irradiation [28]. A possible reason is that γ-irradiation is a strong mutagenic factor [32]. It can be theorized that the difference in physiological status of embryos between hulled and hull-less lines (observed in the present work) may cause not only early but also late effects during seed germination.

To summarize the experimental data, we conducted a comparison of 22 response reactions to two stress factors. It turned out that for γ-irradiation (previously published data), there were seven ranges of radiosensitivity in the analyzed barley lines [28], and for salt solutions, eight ranges of salt tolerance were identified (see Table 4 and Table 5). There were five common and five unique effects of the two stressors. Among the common effects, 60% were found to occur twice as often after irradiation than after salt stress; 20% of effects occurred twice as often after exposure to salt stress than after irradiation, and one effect was evenly distributed between the two stressors. As a result of a pairwise comparison of effects of γ-irradiation and the salt stressor, it was found that the resistance of KO lines matches that of the WT only by 36.4%. A comparison of responses between *win1* KO and *nud* KO lines revealed 72.7% of similar responses to the two stress factors. At the same time, in an article by Vera Pozolotina [85], it was demonstrated that the seed progeny of dandelion (*Taraxacum officinale* s.l.), which is resistant to chemical pollution, is also radioresistant. Similar conclusions (associated resistance levels toward different stressors) were made in experiments on the impact of drought on the ability of wheat and rye to adapt to low temperatures [86].

## 4. Materials and Methods

### 4.1. A Short Literature Review and Database Compilation

For a brief overview, a bibliography search was conducted using the keywords “*Hordeum vulgare*”, “hulled barley”, “naked barley”, “salt stress”, and “Golden Promise” on Google Scholar, Scopus, and PubMed websites. These platforms aggregate conference abstracts, articles, and scientific books on their official webpages, followed by abstracts and full texts. Simultaneous use of multiple databases was motivated by their different specializations [87]. Additionally, lists of references were examined in publications on related topics.

### 4.2. Plant Models

Previously, two-row spring barley (*H. vulgare* L.) cv. GP has been chosen as donor material for targeted mutagenesis (inactivation) of genes *HvNUD* [13] and *HvWIN1* [14] by means of the Cas9/gRNA system. In the present work, to assess salt tolerance of the obtained KO barley plants, three *nud* KO lines (*nud 01-4*, *nud 05-4*, and *nud 07-1*) and three *win1* KO lines (*win1 17-4-14*, *win1 25-2-2*, and *win1 25-2-18*) were utilized, each carrying different mutation types (deletion, insertion, or their combinations). All the chosen lines possess the same phenotype consistent with an inactivated gene. Namely, all *nud* KO lines have the naked grain [13], and all *win1* KO lines are deficient in epicuticular wax [14]. The KO lines used in the current study have been published previously [28]. An unmodified line of hulled barley cultivar GP served as the WT line (control).

### 4.3. Greenhouse Growth Conditions

Seeds for the experiments were obtained from plants grown in the greenhouse complex of the Institute of Cytology and Genetics, the Siberian Branch of the Russian Academy of Sciences (ICG SB RAS, Novosibirsk, Russia) at the multi-access center Laboratory of Artificial Plant Cultivation at 20–25 °C under a 12 h photoperiod at ~25,000 lux illumination until mature seeds formed. Halogen lamps were used as a light source.

### 4.4. Experimental Design

Salt tolerance of seeds of barley KO lines was assessed under laboratory conditions. According to an analysis of published data [59,88,89], a NaCl solution was used at a concentration of 50, 100, or 150 mM (JSC “Chimreactivsnab”, Ufa, Russia). To evaluate salt tolerance of seed progeny from the barley KO lines, into disposable sterile plastic Petri dishes with a double cotton filter, 10 mL of either distilled water (control grains) or a NaCl solution was added at a concentration of 50–150 mM (stressed groups of grains); 16 seeds were sown in each dish, arranged in the 4 × 4 seed pattern. The Petri dishes were sealed with adhesive tape to prevent water evaporation. The experiments were conducted with three biological replicates, involving a total of 1344 seeds. A comprehensive set of 13,338 measurements and entries was recorded into the database.

### 4.5. Seedling Emergence Rates and Growth Characteristics

The first 2 days of germination were synchronized via keeping of the seeds (planted in Petri dishes) at 6 °C in the dark. Then, the seeds were germinated in a climate room of the Institute of Plant and Animal Ecology, the Ural Branch of the Russian Academy of Sciences (IPAE UB RAS, Ekaterinburg, Russia), at 20 °C under a 12 h photoperiod. The Petri dishes were randomized daily. 

Five days after the sowing of the seeds, 20 parameters were quantified: (1) seed germination, seedling survival, and the number of seedlings with leaves; (2) growth parameters: shoot length, the average number of roots, lengths of roots 1 to 11 [root test], seedling length, shoot weight, root weight, and seedling weight.

Seed germination was determined by means of the formula:

(1) $$G,\%=\frac{N_{G}}{N_{T}}\times 100,$$

where *N*_G_ is the number of germinated seeds, and *N*_T_ denotes the total number of sown seeds.

Seedling survival was computed as follows:

(2) $$S,\%=\frac{\left(N_{G}-N_{D}\right)}{N_{T}}\times 100,$$

where *N*_G_ is the number of germinated seeds, *N*_D_ represents the number of dead seedlings, and *N*_T_ denotes the total number of sown seeds.

The number of seedlings with leaves was determined as

(3) $$L,\%=\frac{N_{G}}{N_{T}}\times 100,$$

where *N*_G_ is the number of seedlings with leaves, and *N*_T_ stands for the total number of sown seeds. There were three biological replicates for the determination of the above parameters.

Shoot length of each seedling was measured using a metal ruler with an accuracy of 1 mm [90]. The length of each (from the first to the eleventh) root of the seedlings [root test] was measured also using a metal ruler with an accuracy of 1 mm, too. For root length and shoot length determination, each seedling was a biological replicate. The roots were separated from the shoots, air-dried completely, and weighed to the nearest 1 μg. We calculated the average number of roots, the total length of the root system, the average root length of seedlings, and the shoot/root length ratio. 

To assess variation of a trait, the coefficient of variation (*CV*) was computed using the formula:

(4) $$CV,\%=\frac{SD}{M}\times 100,$$

where *SD* is standard (mean square) deviation, and *M* stands for an arithmetic average value. 

The calculated *CV*s were utilized to rank the levels of variation evaluated according to Prof. Stanislav A. Mamaev’s classification [91]: very low (*CV* < 7%), low (*CV* = 8–12%), moderate (*CV* = 13–20%), increased (*CV* = 21–30%), high (*CV* = 31–40%), and very high (*CV* > 40%) variation.

### 4.6. Estimation of Seedlings’ Abnormalities 

The various abnormalities in the development of seedlings (alterations in the root shape, root pubescence, root necrosis, changes in the leaf color and leaf shape, alterations in coleoptile shape, and the appearance of twins) were estimated visually in each seedling. The frequency of occurrence of each abnormality was calculated as

(5) $$A,\%=\frac{N_{A}}{N_{S}}\times 100,$$

where *N_A_* is the number of instances of an abnormality in a Petri dish, and *N*_S_ is the number of surviving seedlings. There were three biological replicates for the above parameters.

### 4.7. Salt Tolerance Index

This index was determined according to the criteria described above, in relative units toward a respective no-treatment control:

(6) $$STI,\%=\frac{P_{S}}{P_{C}}\times 100$$

where *P_S_* is a parameter’s value under salt stress conditions, and *P_C_* denotes a parameter’s value under control conditions.

### 4.8. Data Analysis

The determined growth parameters of the seedlings were normalized to the time points of measurements in order to minimize variance among replicates and different lines. The normality of the data distribution was evaluated by the Kolmogorov–Smirnov test (*d*) with Lilliefors and Shapiro–Wilk (*W*) corrections. Statistical hypotheses were assessed by the asymptotic two-sided test for a difference between two proportions, by the Kruskal–Wallis (*H*) test, ANOVA, MANOVA, Dunn’s multiple comparison test, and correlation (*R*) analyses. The calculations were carried out in STATISTICA 10.0 [92] software.

## 5. Conclusions

Potential pleiotropic effects of KOs of *HvNUD* and of *HvWIN1* on the resistance of barley seedlings to the salt stressor were detected successfully. It was confirmed that under unstressful (normal) conditions, the KO lines did not significantly differ from the WT in seed germination, seedling survival, and the number of seedlings with leaves. An increase in salt tolerance was noted in lines with the KO of the *HvNUD* gene as compared to the WT in terms of root and shoot length and in weights of roots and shoots. The emergence of root necrosis in *nud* KO lines may be explained by damage or stress to the obtained embryos during grain processing and storage. Lines with a KO of the *HvWIN1* gene under stressful conditions demonstrated the preservation of root length as compared to unstressful conditions and stable variation of most of morphological traits, without correlations between growth magnitudes of different organs before and after exposure to NaCl solutions.

Under the influence of the salt stressor, morphological abnormalities were identified in *win1* KO lines, manifested as changes in the leaf shape and color and the appearance of twins. Overall, for most of the studied parameters, salt tolerance of the analyzed barley lines can be ranked as follows: *nud* KO lines > *win1* KO lines ≈ WT.

A comparison of effects of the salt stressor (current study) and γ-irradiation [28] on KO lines (*nud* and *win1)* indicates differences in barley responses to the two stressors. The tolerance of the KO lines to the two stress factors matched that of the WT by 36.4%, whereas for the comparison of *win1* KO lines with *nud* KO lines, the match was 72.7%. It is possible that tolerance of these lines also differs to other abiotic factors.

## Figures and Tables

**Table 1:** Coefficients of variance (*CV*, %) for key morphometric indicators of seedlings of the seven barley lines under salt stress.

Lines	NaCl Concentration, mM				Fold Increase in *CV* at 150 mM Compared to 0 mM
	0	50	100	150	
Average shoot length					
WT	22.81	30.00	37.75	69.15	3.0 ↑
*nud 07-1*	23.28	24.61	21.62	44.13	1.9 ↑
*nud 05-4*	28.36	27.46	36.50	50.07	1.8 ↑
*nud 01-4*	18.58	19.67	22.65	23.09	1.2 ↔
*win1 25-2-18*	21.52	19.08	18.65	26.87	1.2 ↔
*win1 25-2-2*	12.61	19.06	24.56	20.24	1.6 ↑
*win1 17-4-14*	21.56	17.60	27.23	30.68	1.4 ↑
Average root length					
WT	29.24	36.65	28.85	29.39	1.0 ↔
*nud 07-1*	43.87	25.34	20.68	22.65	0.5 ↓
*nud 05-4*	25.36	29.24	28.20	24.56	1.0 ↔
*nud 01-4*	30.52	26.35	23.98	27.07	0.9 ↔
*win1 25-2-18*	26.01	17.17	16.73	24.78	1.0 ↔
*win1 25-2-2*	25.83	21.52	27.61	22.65	0.9 ↔
*win1 17-4-14*	27.29	27.91	30.90	24.97	0.9 ↔
Shoot length/Average root length					
WT	27.52	26.74	33.45	81.90	3.0 ↑
*nud 07-1*	35.19	18.71	17.65	35.90	1.0 ↔
*nud 05-4*	37.21	14.89	26.42	36.46	1.0 ↔
*nud 01-4*	30.13	13.02	10.40	15.50	0.5 ↓
*win1 25-2-18*	15.26	12.32	16.14	21.62	1.4 ↑
*win1 25-2-2*	15.72	21.86	19.41	21.54	1.4 ↑
*win1 17-4-14*	24.63	15.42	25.21	23.74	1.0 ↔
Shoot length/Sum of root lengths					
WT	29.65	28.90	32.42	104.17	3.5 ↑
*nud 07-1*	39.58	22.37	19.30	38.37	1.0 ↔
*nud 05-4*	32.78	17.32	23.51	53.74	1.6 ↑
*nud 01-4*	32.36	15.29	14.52	15.10	0.5 ↓
*win1 25-2-18*	48.72	13.31	19.12	22.29	0.5 ↓
*win1 25-2-2*	17.25	28.16	18.13	23.11	1.3 ↑
*win1 17-4-14*	27.32	21.24	25.35	22.23	0.8 ↔
Seedling length (shoot + root)					
WT	27.39	40.45	40.41	43.71	1.6 ↑
*nud 07-1*	31.43	23.99	22.00	32.10	1.0 ↔
*nud 05-4*	27.94	29.70	34.69	38.35	1.4 ↑
*nud 01-4*	25.37	29.55	26.83	31.22	1.2 ↔
*win1 25-2-18*	24.18	16.87	16.61	25.96	1.1 ↔
*win1 25-2-2*	26.50	17.19	28.62	27.07	1.0 ↔
*win1 17-4-14*	25.01	29.39	35.67	28.80	1.2 ↔
From first to sixth root length *					
WT	31.3 (27.9–38.1)	37.2 (33.8–39.3)	30.8 (28.6–33.1)	33.2 (27.2–39.0)	0.9–1.2 ↔
*nud 07-1*	48.4 (33.8–51.9)	27.8 (22.0–35.1)	24.7 (18.2–32.7)	28.5 (23.4–33.6)	0.5–0.7 ↓
*nud 05-4*	29.5 (22.3–36.7)	30.8 (29.1–35.0)	29.7 (24.4–35.9)	28.3 (25.9–32.7)	0.7–1.3 ↓ ↑ ↔ **
*nud 01-4*	35.5 (29.9–38.6)	26.1 (20.3–39.9)	26.2 (21.6–34.9)	24.6 (15.6–34.5)	0.4–1.2 ↓ ↔ **
*win1 25-2-18*	27.6 (23.9–39.0)	19.1 (16.3–24.9)	20.0 (17.3–28.2)	25.5 (21.7–32.5)	0.8–1.1 ↔
*win1 25-2-2*	25.7 (21.7–34.0)	25.4 (19.8–38.8)	26.2 (23.4–31.6)	24.8 (22.0–32.0)	0.8–1.1 ↔
*win1 17-4-14*	31.0 (26.1–40.6)	28.7 (24.9–36.8)	27.7 (22.9–34.0)	28.7 (23.5–37.1)	0.9–1.1 ↔

Changes in parameters at the highest NaCl concentration (150 mM) as compared to control values (0 mM) are indicated by arrows. ↔: close to control values, ↓: below control values, ↑: above control values. * Data on six roots are provided: mean *CV* (minimum–maximum). Data on the *CV* for the length of the 7th root are not presented due to the absence of the 7th root in some lines. ** Directions of effects were different between lines *nud 05-4* and *nud 01-4* in terms of 1–6 roots. The fold increase in the *CV* is provided only for group “150 mM NaCl” because this concentration yielded the strongest effect; *n* = 41–48.

**Table 2:** The distribution of seedling abnormalities among naked (*nud 07-1*, *nud 05-4*, and *nud 01-4*) and hulled (WT, *win1 25-2-18* KO, *win1 25-2-2* KO, and *win1 17-4-14* KO) barley lines under salinity stress.

Groups	Abnormalities
Present in all lines	Root hairiness
	Change in coleoptile shape
	Change in leaf color
Absent in all lines	Change in coleoptile color
Rarely found in WT	Change in shape of first root
Present in *nud* KO and *win1* KO lines	Change in leaf tip color
	Necrosis of one root
	Dancer (multiple twists of roots)
	Change in shape of second root
	Change in shape of third root
	Change in leaf shape
Present only in *nud* KO lines	Necrosis of two roots
	Necrosis of three roots
	Necrosis of four roots
	Necrosis of five roots
	Necrosis of six roots
	Necrosis of all roots
Present only in one *win1* KO line (*win1 25-2-18*)	Twins

**Table 3:** A short review of salt tolerance of hulled and naked barley cultivars, as assessed by means of different physiological (morphometric) parameters.

Cultivars, Varieties	Grains	Organs	Endpoints	NaCl, mM	Exposure	Age	Effects	Salt Tolerance	Reference
GP, Maythorpe	Hd	Shoots	FW	150	4 w	7 w	↓	GP < PP Maythorpe	[44]
GP, Maythorpe	Hd	Roots	FW	150	4 w	7 w	↓	GP > PP Maythorpe	[44]
GP, Maythorpe	Hd	Grains	GW	25 (control) 150	4 w	unspecified	↓	GP > PP Maythorpe	[45] quoted from [39]
GP, Maythorpe	Hd	Plants	PW	25 (control) 150	4 w	unspecified	↓	GP > PP Maythorpe	[45] quoted from [39]
GP, Maythorpe	Hd	Plants	PH	25 (control) 150	4 w	unspecified	↓	GP < PP Maythorpe	[45] quoted from [39]
GP, Maythorpe	Hd	Plants	NS	25 (control) 150	4 w	unspecified	↓	GP > PP Maythorpe	[45] quoted from [39]
GP, Maythorpe	Hd	Plants	DW	50 100 150	2–6 d	52 d	↔ ↓ ↓	GP > PP Maythorpe GP > PP Maythorpe GP ≈ PP Maythorpe	[42]
Clipper, Skiff, Keel, Barque, Mundah, ST: CM72, Sahara, PI71284-48	Hd	Roots	RL	100 150	unspecified	unspecified	↓ ↓	–	[46]
Clipper, Skiff, Keel, Barque, Mundah, ST: CM72, Sahara, PI71284-48	Hd	Shoots	DW	100 150	unspecified	unspecified	↓ ↓	–	[46]
GP, Numar, Naso	Hd	Shoots	FW, DW	150	4 w	5 w	↓	Numar < GP < Naso	[40]
GP, XZ113, H559	Hd	Shoots	FW, DW	400	7 d	17–27 d	↓	GP < XZ113 < H559	[41]
GP, XZ113, H559	Hd	Roots	FW, DW	400	7 d	17–27 d	↓	GP < XZ113 < H559	[41]
PI 219796, PI 254894, PI 268243, PI 296843, PI 560558	Hd	Roots	RL	171, 257, 342	10 d	10 d	↓	–	[47]
PI 219796, PI 254894, PI 268243, PI 296843, PI 560558	Hd	Shoots	SL	171, 257, 342	10 d	10 d	↓	–	[47]
PI 219796, PI 254894, PI 268243, PI 296843, PI 560558	Hd	Seeds	G	342	10 d	unspecified	=0	–	[47]
GP	Hd	Roots	RL	200	10 d	22 d	↓	–	[48]
Naked barley	Nd	Seedlings	PH	30 60, 90, 120	4–6 d	4–6 d	↔ ↓	–	[49]
Naked barley	Nd	Roots	RL	30 60, 90, 120	4–6 d	4–6 d	↔ ↓	–	[49]
Naked barley	Nd	Seedlings	FW	30 60, 90, 120	6 d	6 d	↔ ↓	–	[49]
Naked and hulled barley	Nd, Hd	Grains	G	171 342	unspecified ^a^	unspecified ^a^	↓	Nd > Hd	[50]
Naked and hulled barley	Nd, Hd	Roots	RL	171 342	unspecified ^a^	unspecified ^a^	^a^	Nd > Hd	[50]
Naked and hulled barley	Nd, Hd	Shoots	SL	171 342	unspecified ^a^	unspecified ^a^	^a^	Nd > Hd	[50]
Özen, Tarm	Nd, Hd	Roots	RL	DHS (−0.05 MPa, PEG 6000 + 150 mM NaCl + 35 °C)	24–72 h	2 w	↓	Nd < Hd	[43]

Abbreviations: DW: dry weight, FW: fresh weight, G: germination, GP: Golden Promise, GW: grain weight, Hd: hulled barley, Nd: naked barley, NS: the number of seeds per spike, PH: plant height, PP: parent cultivar, PW: plant weight, RL: root length, SL: shoot length, ↓: below control values, ↔: close to control values, ^a^ full text of the article is not available online.

**Table 4:** Comparative evaluation of significant differences among barley KO lines in terms of seedling survival, root and shoot parameters, and abnormalities under the action of the salt stressor.

Parameters	Salt Tolerance Range	Resume
Seedling survival and root and shoot parameters		
Seed germination	*win1* < *WT* < *nud*	*win1* KO is more sensitive
Survival rate	*win1* < *nud* < *WT*	*win1* KO is more sensitive
Seedlings with leaves	*WT* < *nud* < *win1*	WT is more sensitive
Shoot length	*WT* < *nud* < *win1*	WT is more sensitive
Mean root length	*win1* < *WT* < *nud*	*win1* KO is more sensitive
Total root length	*WT* ≈ *win1* < *nud*	*nud* KO is more tolerant
Seedling length	*WT* < *win1* < *nud*	WT is more sensitive
Root number	*WT* < *nud* < *win1*	WT is more sensitive
First root length	*WT* ≈ *win1* < *nud*	*nud* KO is more tolerant
Second root length	*WT* ≈ *win1* < *nud*	*nud* KO is more tolerant
Third root length	*win1* < *WT* < *nud*	*win1* KO is more sensitive
Fourth root length	*win1* < *WT* < *nud*	*win1* KO is more sensitive
Fifth root length	*WT* ≈ *win1* < *nud*	*nud* KO is more tolerant
Sixth root length	*WT* ≈ *win1* < *nud*	*nud* KO is more tolerant
Seventh root length	*WT* < *win1* < *nud*	WT is more sensitive
Shoot/Mean root length	*nud* < *win1* ≈ *WT*	*nud* KO is more sensitive *
Shoot/Total root length	*nud* < *win1* < *WT*	*nud* KO is more sensitive *
Root weight	*win1* < *WT* < *nud*	*win1* KO is more sensitive
Shoot weight	*WT* ≈ *nud* < *win1*	*win1* KO is more tolerant
Seedling weight	*WT* ≈ *win1* < *nud*	*nud* KO is more tolerant
Single-seedling weight	*WT* < *nud* < *win1*	WT is more sensitive
Morphological abnormalities		
Hairy roots	*win1* < *nud* < *WT*	*win1* KO is more sensitive

Similar stress tolerance patterns are highlighted in one color. * Because *nud* KO lines exhibit hormetic effects in terms of root growth under the stressful conditions (denominator becomes larger), the shoot/root length ratio decreases. In fact, *nud* KO lines are salt-tolerant.

**Table 5:** A distribution (for WT and KO barley lines) of frequencies (%) of reactions to the salt stressor and to γ-irradiation.

Tolerance Range	Stressors	
	Salt	γ-Irradiation
*nud* < *win1* < *WT*	4.55	9.09
*nud* < *win1* ≈ *WT*	4.55	–
*win1* < *nud* < *WT*	9.09	18.18
*win1* < *WT* < *nud*	22.73	45.45
*win1* < *WT* ≈ *nud*	–	4.55
*win1* ≈ *nud* < *WT*	–	9.09
*WT* < *nud* < *win1*	18.18	4.55
*WT* < *win1* < *nud*	9.09	9.09
*WT* ≈ *nud* < *win1*	4.55	–
*WT* ≈ *win1* < *nud*	27.27	–

## Data Availability

Raw data are available upon request.
